# CSDE1: a versatile regulator of gene expression in cancer

**DOI:** 10.1093/narcan/zcae014

**Published:** 2024-04-10

**Authors:** Annagiulia Ciocia, Neus Mestre-Farràs, Ignacio Vicent-Nacht, Tanit Guitart, Fátima Gebauer

**Affiliations:** Centre for Genomic Regulation (CRG), The Barcelona Institute of Science and Technology, Dr Aiguader 88, Barcelona 08003, Spain; Universitat Pompeu Fabra (UPF), Dr Aiguader 88, Barcelona, Spain; Centre for Genomic Regulation (CRG), The Barcelona Institute of Science and Technology, Dr Aiguader 88, Barcelona 08003, Spain; Centre for Genomic Regulation (CRG), The Barcelona Institute of Science and Technology, Dr Aiguader 88, Barcelona 08003, Spain; Universitat Pompeu Fabra (UPF), Dr Aiguader 88, Barcelona, Spain; Centre for Genomic Regulation (CRG), The Barcelona Institute of Science and Technology, Dr Aiguader 88, Barcelona 08003, Spain; Centre for Genomic Regulation (CRG), The Barcelona Institute of Science and Technology, Dr Aiguader 88, Barcelona 08003, Spain; Universitat Pompeu Fabra (UPF), Dr Aiguader 88, Barcelona, Spain

## Abstract

RNA-binding proteins (RBPs) have garnered significant attention in the field of cancer due to their ability to modulate diverse tumor traits. Once considered untargetable, RBPs have sparked renewed interest in drug development, particularly in the context of RNA-binding modulators of translation. This review focuses on one such modulator, the protein CSDE1, and its pivotal role in regulating cancer hallmarks. We discuss context-specific functions of CSDE1 in tumor development, its mechanisms of action, and highlight features that support its role as a molecular adaptor. Additionally, we discuss the regulation of CSDE1 itself and its potential value as biomarker and therapeutic target.

## Graphical Abstract

**Graphical Abstract:**
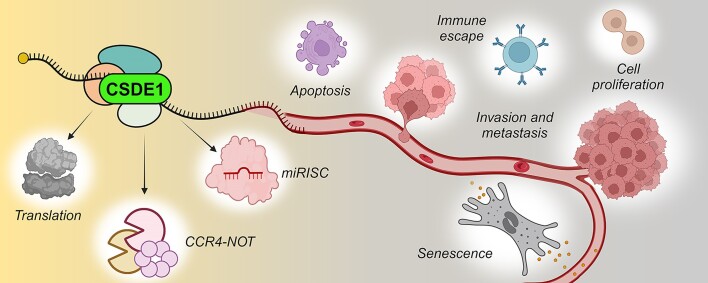

## Introduction

RNA-binding proteins (RBPs) constitute one of the most highly conserved and largest protein families in the cell (1,2). They modulate all steps in the gene expression cascade, orchestrating RNA fate and function. RBPs can bind hundreds of RNAs, forming extensive regulatory networks that help to maintain cell homeostasis (3–5). Canonical RBPs possess structured RNA-binding domains (RBDs) that establish well-defined molecular interactions with RNA, facilitating the computational prediction of RBP–RNA interactions (6). Recent high-throughput technologies, however, have revealed a plethora of unconventional RBPs which lack defined RBDs and often interact with RNA using disordered regions (2,7–9). While canonical RBPs show relatively low rates of mutation in cancer, unconventional RBPs seem to be highly mutated in proliferation disorders, although their roles need to be further investigated (10–12). Irrespective of their mutation rate, RBPs have the capacity to modulate virtually all cancer hallmarks and underlie adaptive mechanisms of stress resistance, ultimately promoting cell plasticity and cancer aggressiveness (13–15). In addition, RBPs are gaining recognition as crucial regulators of therapy resistance and immunomodulation, sparking a growing interest in targeting RBPs for cancer prevention and treatment. Targeting options are diverse, and include small molecule inhibitors, antisense oligonucleotides, small interfering RNAs, nucleic acid or protein aptamers, small peptides and RNA-PROTACS (16–21).

One of the gene expression steps in which RBPs play important regulatory roles is mRNA translation (22). Regulation of translation quickly impacts cell protein content and can rapidly modulate cell behavior upon environmental challenges, such as the numerous hurdles a cancerous cell must overcome to successfully colonize distant organs. Multiple routes contribute to translational reprogramming of cancer cells, including—but not limited to—alterations in the levels or activity of translation factors, fluctuations in tRNA abundance, epigenetic modifications within the ribosome or transcriptome, and variations in trans-acting regulators such as miRNAs or RBPs (extensively reviewed in 20,23–25). Together, these mechanisms cooperate to enhance the translation of specific mRNA subsets within cancer cells, shaping their behavior in response to intrinsic and extrinsic signals.

This review centers on Cold Shock Domain-containing E1 (CSDE1), also known as Upstream of N-Ras (UNR), a highly conserved RBP that regulates mRNA translation and stability. CSDE1 is essential for embryonic development, as disruption of CSDE1 in mice causes defects in neural tube and placenta formation leading to lethality at mid-gestation (26). CSDE1 plays a crucial role in stem cell regulation by inhibiting the differentiation of both mouse and human embryonic stem cells toward specific lineages and, in zebrafish, by promoting the formation of hematopoietic stem and progenitor cells (27–29). CSDE1 has also been reported to control neural cell migration (30,31). Perhaps related to these functions, CSDE1 mutations have been associated with a number of neurodevelopmental and adult-onset psychiatric disorders including autism, intellectual disability, and psychosis (32–35). Furthermore, CSDE1 has been linked to blood disorders and cardiovascular disease (36–38). Here, we focus on the role of CSDE1 in the cancer context. We first summarize the literature linking CSDE1 with cancer development and highlight cancer hallmarks regulated by CSDE1. We then focus on molecular features and mechanisms that allow CSDE1 to perform its functions, review how CSDE1 itself is regulated, and conclude with our perspective on CSDE1 as an emerging therapeutic target.

## Cancer hallmarks regulated by CSDE1 and mechanisms of action

**Figure 1.**
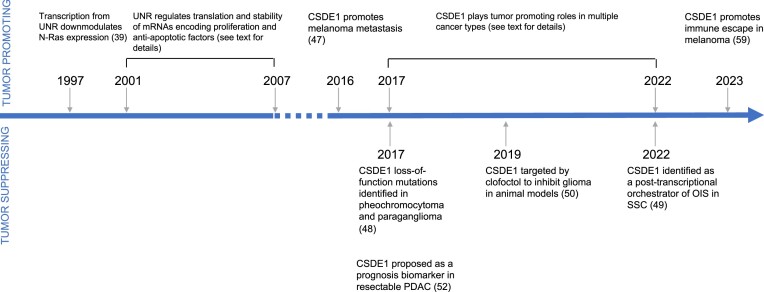
A brief history of CSDE1 in cancer. Key reports supporting tumor promoting or suppressing roles of CSDE1 are indicated above or below the arrow, respectively. References are indicated in parenthesis. See text for more details.

The CSDE1 locus maps approximately 150 bp upstream of the N-Ras gene in the mammalian genome (hence the alternative name of UNR). A potential link of CSDE1 with cancer dates back to 1997, when evidence of transcriptional interference between UNR and the N-Ras locus was reported (39) (Figure 1). In the following few years, data accumulated suggesting a role of CSDE1 in cell proliferation and survival via regulation of important proto-oncogenes, anti-apoptotic factors and cell cycle kinases. CSDE1 was identified as an IRES trans-acting factor (ITAF), promoting cap-independent translation of c-MYC, APAF-1 and CDK11^p58^ transcripts (40–43). Molecularly, at least in the case of APAF-1, CSDE1 behaved as an RNA chaperone, modulating the structure of the IRES together with PTB in order to create a single stranded region for ribosome landing (41). In addition, CSDE1 was found to stabilize c-FOS mRNA by binding to a region in its coding sequence called ‘major coding region determinant’ (mCRD) and protecting the poly(A) tail from attack by CCR4 together with PABPC1 (44,45). Other reports confirmed a role of CSDE1 in protecting cells from apoptosis, although the molecular mechanisms were not investigated (46). It was not until 2016, however, that CSDE1 was identified as a *bona fide* oncoprotein (47). Depletion of CSDE1 from melanoma cells dramatically decreased metastasis in mice, whereas CSDE1 over-expression in a non-metastatic cell line markedly increased its metastatic potential. The underlying mechanism was attributed to coordinated control of mRNA regulons, with a central role played by CSDE1-mediated stimulation of translation elongation of VIM and RAC1 mRNAs, involved in epithelial-to-mesenchymal (EMT) transition (47). Since then, research on CSDE1 has rapidly grown, revealing context-specific roles of this protein in cancer. In 2017, Fishbein *et al.* found CSDE1 loss-of-function mutations as drivers of neuroendocrine tumors with a Wnt-altered phenotype, indicating that CSDE1 is a suppressor of this tumor type (48). CSDE1 also behaves as a tumor suppressor in squamous cell carcinoma, where it promotes oncogene-induced senescence by repressing the synthesis of YBX1 and enhancing the stability of mRNAs encoding senescence-associated secretory phenotype (SASP) factors (49). In addition, tumor suppressor roles of CSDE1 can be inferred from studies in glioma, where CSDE1 was identified as the target of the drug clofoctol (50). Clofoctol binds to CSDE1 and enhances its association with KLF13 mRNA -encoding a tumor suppressor- leading to KLF13 mRNA stabilization, reduction of tumor growth and increased mouse survival. Importantly, these studies highlight the potential of CSDE1 as a druggable target. Although these findings support a beneficial role of CSDE1 in glioma, a conflicting report suggests that CSDE1 promotes glioma cell migration and is significantly upregulated in patient samples compared to normal brain tissues (51). Likewise, contradictory results have been reported for pancreatic adenocarcinoma (PDAC). Martinez-Useros *et al.* (2017) proposed CSDE1 as a biomarker associated with favorable prognosis in resectable PDAC (52). However, this contrasts with functions of CSDE1 in promoting invasion and clonogenicity in a number of PDAC cell lines *in vitro* (53). These results suggest complex behaviors of CSDE1 in some tumor types. Nevertheless, in most examples studied to date CSDE1 performs tumor promoting roles. These include melanoma, colorectal, breast, thyroid and lung cancers, among others (54–57). The molecular mechanisms that CSDE1 employs to promote tumor formation in these cancer types remain largely elusive. An exception is melanoma, where CSDE1 behaves as a versatile ‘Swiss knife’ to promote an invasive phenotype. In addition to translational upregulation of EMT markers mentioned above, CSDE1 attenuates miRNA-mediated silencing of the TGF-ß signaling regulator PMEPA1 in melanoma, and maintains the levels of ß-catenin likely by promoting its translation, as shown in hematopoietic cells (29,47,58). Furthermore, an elegant recent report showed that CSDE1 promotes immune escape in melanoma by stabilizing the levels of PTPN2 mRNA, which encodes the STAT1 phosphatase TCPTP (59). Hypo-phosphorylated STAT1, in turn, cannot activate the transcription of genes involved in class I antigen presentation, leading to immune evasion. Notably, the authors showed that epigenetic modification of the CSDE1 promoter by SMYD3 is crucial to increase CSDE1 levels, and proposed the SMYD3-CSDE1 axis as a potential biomarker for immunotherapy response.

**Figure 2.**
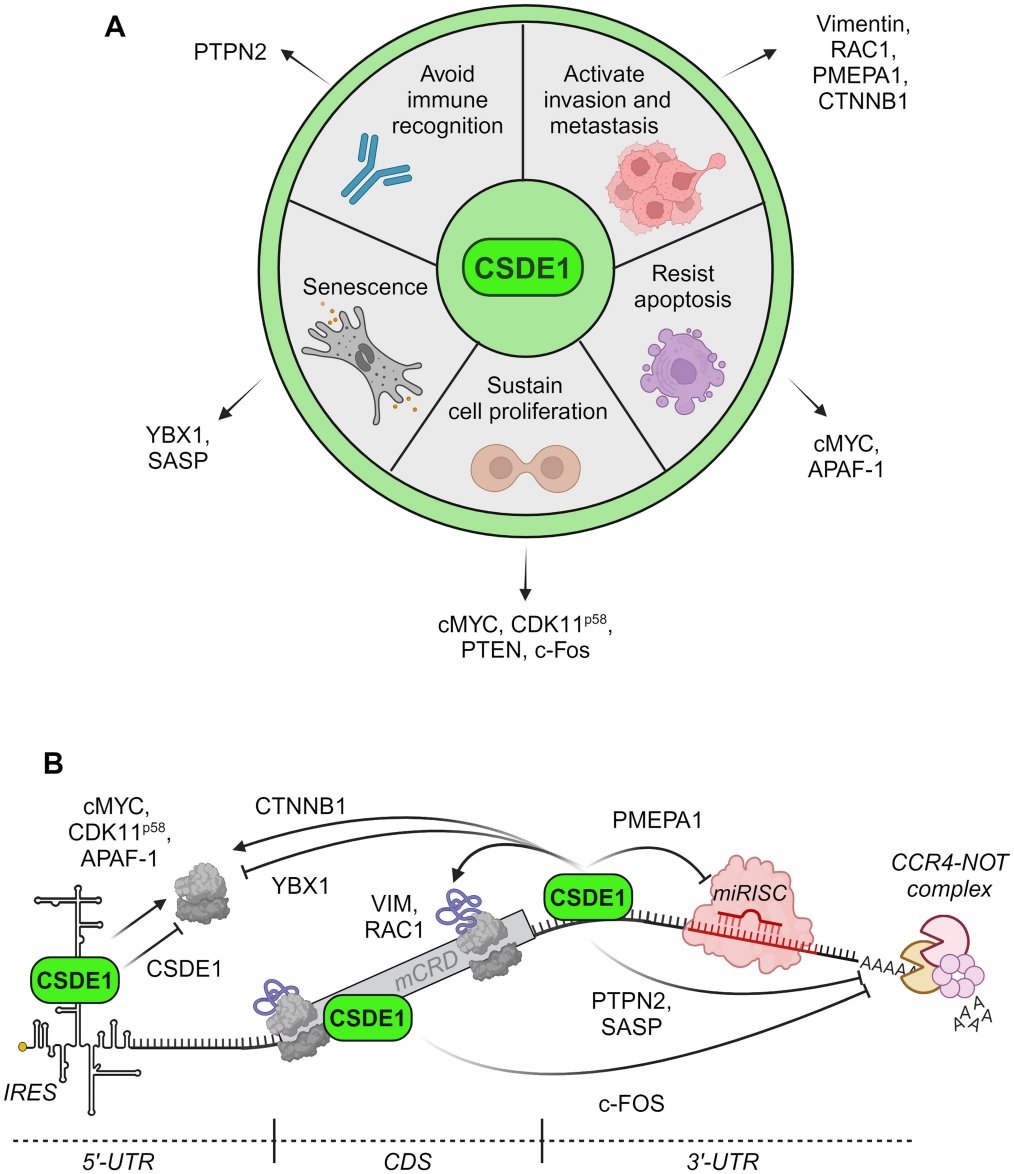
Regulation of cancer hallmarks by CSDE1. (**A**) Cancer hallmarks and mRNA targets regulated by CSDE1. (**B**) Molecular mechanisms of regulation. Mechanisms have been condensed on a hypothetical mRNA. Specific mRNA targets are indicated on the arrows. See text for details. Created with BioRender.com.

In summary, CSDE1 regulates five of the fourteen cancer hallmarks described by Hanahan (60), namely proliferation, survival, invasion, senescence and immune evasion, via regulation of specific mRNA targets (Figure 2A). The molecular mechanisms involve regulation of mRNA translation and/or stability, although details on how CSDE1 interacts with the mRNA translation and degradation machineries are lacking in most cases (Figure 2B). The data, however, clearly show that CDSE1 is a versatile modulator. CSDE1 can inhibit or enhance gene expression depending on the molecular target and the biological context. For example, CSDE1 enhances translation elongation of VIM and RAC1 mRNAs to promote invasiveness, but also represses translation initiation from its own IRES as a feed-back mechanism to control its levels during cell cycle progression (47,61). Similarly, to promote melanoma cell aggressiveness CSDE1 downregulates the levels of PTEN mRNA, while it upregulates the levels of PTPN2 and PMEPA1 mRNAs (47,58,59). The flexibility of CSDE1 to recognize RNA and its capacity to interact with multiple factors probably underlie the plasticity of CSDE1 as a regulator. Indeed, CSDE1 has been proposed to function as a molecular adaptor, a platform for interactions with other molecules that dictate CSDE1 ultimate functions (62). The properties of CSDE1 that may allow this versatility are discussed in the following sections.

## CSDE1 as a molecular adaptor

**Figure 3.**
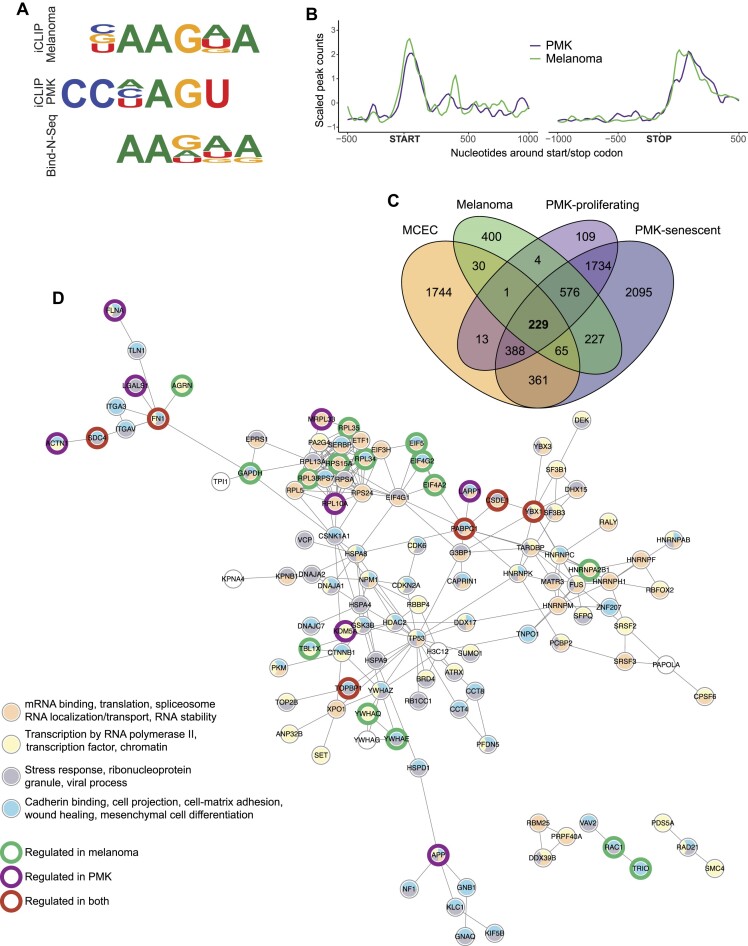
CSDE1 mRNA binding. (**A**) Seqlogo of CSDE1 in two iCLIP experiments, performed in human melanoma SK-Mel-147 cells and primary mouse keratinocytes (PMK), and Bind-N-Seq experiments in cell-free extracts. (**B**) Metaprofiles of CSDE1 binding to its targets in melanoma and PMK. iCLIP peak counts from references (47) and (49) were averaged across replicates and scaled for comparison. (**C**) Venn diagram comparing CSDE1 RNA targets in mouse cardiac endothelial cells (MCEC) (74), melanoma (47) and either proliferating or senescent PMK (49). A core of 229 targets is highlighted. (**D**) Core network of CSDE1 targets. The network was built using STRING with a confidence score of at least 0.7, and the 229 core targets in (C) as input. Edges represent direct binding between two proteins, or that these proteins are part of the same physical complex. Only sub-networks with at least 3 edges are represented. Nodes are colored according to GEO terms. Thick borders highlight targets regulated in cancer. Note that not all targets regulated in cancer are shown.

CSDE1 binding specificity is somewhat relaxed. Initial *in vitro* SELEX experiments indicated a purine-rich motif with a core of AAGUA/G or AACG, which has been confirmed in Bind-N-Seq experiments and *in vivo* iCLIP studies (47,49,63,64) (Figure 3A). While these motifs might be present frequently in the transcriptome, a meta-analysis reveals a predilection for CSDE1 binding near the start codon and following the termination codon of the open reading frame, suggesting that additional features contribute to RNA-binding specificity (Figure 3B). One such feature may be the binding to other proteins. Judging from examples of the CSDE1 homologue in *Drosophila*, it seems clear that binding to RNA and interactions with protein partners mutually influence each other. *Drosophila* UNR and its partner SXL synergistically bind to adjacent sites in *msl-2* mRNA, while UNR binding to the lncRNA *roX2* allows subsequent interactions of UNR with the RNA helicase MLE (65,66). Thus, the selectivity of CSDE1 towards its RNA targets can probably not be understood without the contribution of its protein partners. One factor that appears frequently associated to CSDE1 is PABPC1. The two proteins have been reported to mutually influence each other's RNA binding, with consequences for regulation of mRNA stability and translation (45,67–69). Another frequent partner of CSDE1 is STRAP -also called Unrip (for UNR-interacting protein)- a factor involved in spliceosomal snRNP assembly and regulation of TGF-ß signaling (70–72). High-throughput studies performed in erythroblasts suggest that STRAP can affect the expression of select CSDE1 targets without significantly altering CSDE1 RNA-binding capability (73), but equivalent studies in cancer cells are missing. Unfortunately, no unbiased efforts have been reported to identify partners of mammalian CSDE1 in cancer cells, a gap that must be addressed to fully understand CSDE1 binding and regulation of mRNA targets.

In order to obtain a global view of CSDE1 targets, we compared the transcripts bound to endogenous CSDE1 in three large-scale studies performed in melanoma cells, primary mouse keratinocytes (PMK, either under proliferating or senescent conditions), and mouse cardiac endothelial cells (MCEC) (47,49,74). Although thousands of transcripts were bound by CSDE1 in each condition, a common set of 229 targets was found, supporting the prevalent context-specific nature of CSDE1 interactions with RNA (Figure 3C). The core set of commonly bound transcripts were enriched in functional terms related to stress response, cell migration and (post)-transcriptional regulation, underscoring the relevance of CSDE1 as a modulator of cancer cell behavior (Figure 3D). Notably, a considerable subset of these transcripts code for proteins that are physically connected, highlighting regulons coordinated by CSDE1 (Figure 3D).

It is important to note that CSDE1 binding does not necessarily imply regulation. We usually find that only ∼20% of the CSDE1-bound transcripts are regulated by CSDE1 in any given condition, and this remains true for the core network (Figure 3D). Furthermore, even among targets regulated in several conditions, modulation may occur in different directions (i.e. activation or repression) and through distinct mechanisms. For example, CSDE1 downregulates PTEN mRNA levels in melanoma, while it increases PTEN mRNA translation to prevent squamous cell carcinoma (47,49). In conclusion, CSDE1 binding and regulation of transcripts is highly context-specific. Mounting evidence suggests that this stems from the ability of CSDE1 to serve as a molecular adaptor, wherein protein interactors could influence the selection of mRNA targets by CSDE1 and/or the subsequent molecular mechanisms governing gene expression control.

## CSDE1: structure, isoforms and PTMs

**Figure 4.**
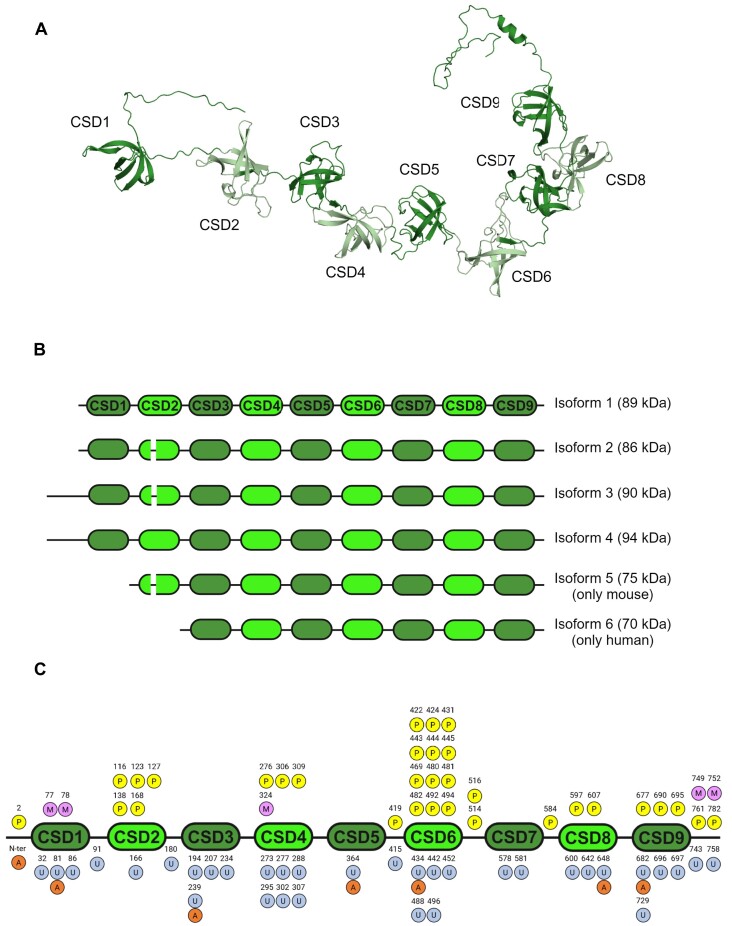
CSDE1 structure, isoforms and PTMs. (**A**) Alphafold rendering of CSDE1 isoform 1 structure showing nine CSDs. URL: https://alphafold.ebi.ac.uk/entry/O75534; Uniprot ID: O75534. (**B**) Isoforms of mammalian CSDE1. Non-canonical CSDs are shown in light green. Isoform number and molecular weights are indicated on the right. Isoforms differ in the N-terminal part containing CSDs 1 and 2. Currently, isoform 5 has only been detected in mouse, and isoform 6 in humans (85). (**C**) CSDE1 PTM landscape. PTMs have been gathered from http://ptm-rbp-atlas.igb.uci.edu (86) and PhosphoSitePlus. Phosphorylation (P, yellow), methylation (M, magenta), ubiquitylation (U, light blue) and acetylation (A, orange) are indicated with the modified amino acid number on top (numbers according to isoform 1). Created with BioRender.com.

CSDE1 contains a string of cold shock domains (CSD), ß-barrel structures that allow binding to single stranded nucleic acids (75) (Figure 4A). Initially, CSDE1 was thought to contain five canonical CSDs, containing RNP motifs that directly contact RNA. A recent report, however, identified four additional CSDs which were termed non-canonical, as they lack RNP motifs and rather influence CSDE1 RNA binding through structural constraints (76). Canonical and non-canonical CSDs alternate in the CSDE1 structure (Figure 4A). Regarding isoforms, more than 50 CSDE1 mRNA species can be found in ENSEMBL, indicating extensive regulation by alternative splicing, alternative transcription start site selection and alternative polyadenylation. These mRNA isoforms collectively give rise to six protein isoforms that differ in their amino-terminal part (Figure 4B). In addition, analysis of public databases indicates that CSDE1 is extensively modified by phosphorylation and ubiquitylation, with a lesser prevalence of acetylation and methylation (Figure 4C). Interestingly, PTMs appear to be more frequently deposited on non-canonical CSDs, suggesting that they may modulate CSDE1 function through structural adjustments. There is currently no information on the individual roles of CSDE1 isoforms, on regulation of CSDE1 mRNA processing, or on the role of post-translational modifications (PTMs) in CSDE1 biology, but we presume that such diversity contributes to the versatile roles of CSDE1 as a molecular adaptor.

## Regulating the regulator

**Figure 5.**
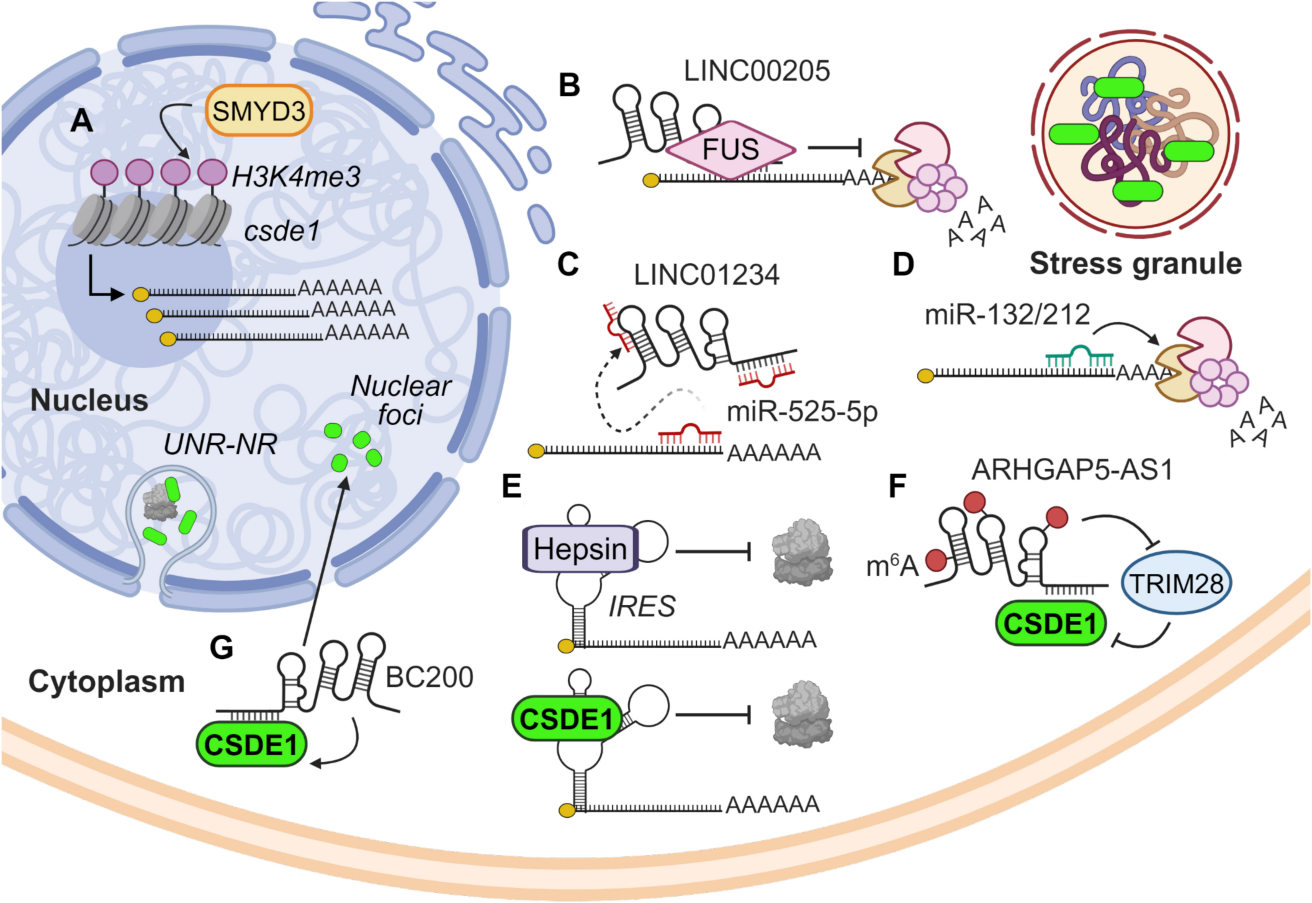
Regulation of CSDE1. Mechanisms of CSDE1 regulation in the nucleus (**A**) and cytoplasm (**B–G**) are indicated. Cellular structures in which CSDE1 has been found in addition to the cytosol are shown. See text for details. Created with BioRender.com.

CSDE1 has been shown to be regulated at many levels of the gene expression cascade, including transcription, mRNA stability, translation, protein turnover and intracellular localization. Epigenetic modification (H3K4me3) of the CSDE1 locus by the methyltransferase SMYD3 controls CSDE1 transcription upon mechanical signals in melanoma nascent tumorigenic cells (59) (Figure 5A). Extensive post-transcriptional regulation at the level of mRNA stability has additionally been reported, often involving both long and short non-coding RNAs. In lung cancer cells, the lncRNA LINC00205 recruits FUS to the CSDE1 transcript, which in turn helps to maintain its stability (56) (Figure 5B). In breast cancer cells, CSDE1 mRNA is targeted by miR-525-5p. LINC01234 acts as a sponge for miR-525-5p, leading to increased proliferation and reduced apoptosis (57) (Figure 5C). CSDE1 mRNA was identified as the target of miR-132/212 in thyroid cancer cells (54) (Figure 5D). Interestingly, CSDE1 is not merely a target of miRNAs, but also participates in miRNA biogenesis and function (77). In erythroid cells, CSDE1 participates in pre-miR-451 processing and trimming in association with AGO2 and PARN (78). Furthermore, in melanoma cells CSDE1 interacts with AGO2 in the miRISC complex and counteracts the silencing of PMEPA1 mRNA by miR-129-5p (58).

Regulation of CSDE1 levels by mechanisms affecting translation and protein stability have been reported. The protease hepsin binds to CSDE1 IRES and inhibits its activity, resulting in reduced CSDE1 translation in prostate cancer cells (79) (Figure 5E). CSDE1 itself negatively regulates translation from its own IRES, tuning CSDE1 levels during the cell cycle (61,80). In fact, autoregulatory feed-back of RBPs is relatively frequent, attesting to their important functions in the cell (81). Regarding protein turnover, an interesting mechanism has been recently reported in hepatocellular carcinoma cells (82). Adenosine methylation (m^6^A) leads to stabilization of the lncRNA ARHGAP5-AS1 which, in turn, binds to CSDE1 and attenuates its interaction with the E3 ubiquitin ligase TRIM28, ultimately leading to reduced CSDE1 proteasomal degradation (Figure 5F).

Finally, CSDE1 localization to specific cellular foci can be regulated. CSDE1 is primarily localized in the cytoplasm of cancer cells. The lncRNA BC200 is involved in preserving this cytoplasmic distribution in breast cancer MCF-7 cells, as depletion of BC200 leads to accumulation of CSDE1 in intranuclear foci and reduced CSDE1 levels (83) (Figure 5G). In addition to its general cytoplasmic distribution, CSDE1 is part of stress granules and is critical for their formation (84). Furthermore, CSDE1 is involved in the formation of specialized structures detected in placental throphoblast cells that consist of cytoplasmic invaginations into the nucleus. These structures have been termed ‘UNR nucleoplasmic reticulum’ or UNR-NR, and are sites of active translation also detected in some cancer cells under stress (26). The role of UNR-NRs in cancer biology remains poorly characterized.

## Concluding remarks

CSDE1 is emerging as an important regulator of cancer cell behavior, and as a flexible molecular adaptor. This RBP can bind to hundreds of mRNAs, and select which ones to regulate and by which mechanism depending on biological context. Over the years, CSDE1 has been identified within gene signatures linked to various cancer types, and more recently it has been proposed as a potential biomarker of prognosis and response to treatment (52,59). In addition, evidence is starting to accumulate that CSDE1 modulation may be useful to enhance drug response (55). CSDE1 itself has been recently identified as the target of Clofoctol, placing CSDE1 in the druggable space (50). Given the context-specific functions of CSDE1 in cancer and our poor understanding of its roles in healthy cells, however, it is crucial to expand our knowledge before considering CSDE1 for therapeutic applications. Many questions remain unanswered: How does CSDE1 recognize its targets and what complexes does it engage with? How is the regulation of CSDE1 RNP complex formation orchestrated? In what manner does CSDE1 interact with the translation and mRNA degradation machineries to govern post-transcriptional gene expression? Do different CSDE1 isoforms perform different functions? How is CSDE1 regulated by post-translational modification? Addressing these questions will allow to delineate critical features that render CSDE1 either a tumor suppressor or an oncogene, and to ultimately devise targeted strategies centered on CSDE1 for precision therapy.

## Data Availability

No new data were generated or analysed in support of this research.
